# Weak Association between Vastus Lateralis Muscle Fiber Composition and Fascicle Length in Young Untrained Females

**DOI:** 10.3390/sports9050056

**Published:** 2021-04-28

**Authors:** Thomas Mpampoulis, Spyridon Methenitis, Constantinos Papadopoulos, Giorgos Papadimas, Polyxeni Spiliopoulou, Angeliki-Nikoletta Stasinaki, Gregory C. Bogdanis, Giorgos Karampatsos, Gerasimos Terzis

**Affiliations:** 1 Sports Performance Laboratory, School of Physical Education & Sports Science, National and Kapodistrian University of Athens, 17237 Athens, Greece; smetheni@phed.uoa.gr (S.M.); spipolyxeni@phed.uoa.gr (P.S.); agstasin@phed.uoa.gr (A.-N.S.); gbogdanis@phed.uoa.gr (G.C.B.); gkarab@phed.uoa.gr (G.K.); gterzis@phed.uoa.gr (G.T.); 2 A’ Neurology Clinic, Aiginition Hospital, Medical School, National and Kapodistrian University of Athens, 11528 Athens, Greece; constantinospapadopoulos@yahoo.com (C.P.); gkpapad@yahoo.gr (G.P.)

**Keywords:** muscle power, rate of force development, fiber type composition, muscle architecture

## Abstract

The aim of the study was to investigate the relationships between vastus lateralis muscle fiber length and fiber type composition in individuals with minimal exposure to systematic resistance/power training. In sixty female physical education students (age: 21.03 ± 2.1 years, body weight: 59.8 ± 9.7 kg, body height: 166.2 ± 6.5 cm), with no experience in systematic training, lean body mass, VL muscle architecture and fiber composition type, countermovement jumping (CMJ) performance, and isometric leg press rate of force development were evaluated. Data were analyzed for all participants, as well as two equally numbered groups assigned according to their maximum countermovement jumping power (High-Power or Low-Power group). Significant but low correlations were found between type II muscle fiber percentage and fascicle length (N = 60, *p* < 0.05). Significant correlations were found between type IIa and IIx muscle fiber percentage cross-sectional area (%CSA) and fascicle length (N = 60; r = 0.321, and r = 0.378; respectively, *p* < 0.05). These correlations were higher for the High-Power group (r = 0.499, and r = 0.522; respectively, *p* < 0.05), and lower, and nonsignificant, for the Low-Power group. The best predictor of strength/power performance was the lean body mass of the lower extremities (r = 0.389–0.645, *p* < 0.05). These results suggest that in females with minimal exposure to systematic training, fascicle length may be weakly linked with type II fiber areas, only in females with high-power profiles.

## 1. Introduction

Muscle power depends on several biological attributes, including muscle fiber composition and muscle fascicle length. An increased proportion and size of fast contracting type II muscle fibers has been linked with higher power performance [1,2], due to the intrinsic contractile characteristics of these muscle fibers [3]. Muscle and tendon morphology and tendon compliance also have a significant contribution to muscle strength and power. For example, tendon stiffness is associated with increased power performance [4]. Muscle thickness is closely linked with the size and anatomical cross-sectional area of muscles, and thus it can be used to evaluate the size and/or the training induced hypertrophy of muscle [5]. Along this line, individuals with greater muscle thickness tend to have a higher maximum strength and power production compared to individuals with smaller muscle thickness, while training-induced changes in muscle thickness are linked to the concomitant increases of maximum strength and power performance [5,6,7,8,9,10,11]. Muscle fascicle pennation angle, e.g., the angle between muscle fascicles and aponeurosis or muscle line action [8], has also be linked with strength [12,13,14], while training-induced changes in pennation angle and maximum strength are closely linked [9], as this influences the mechanical efficiency of force transmission to the tendons/aponeuroses [8,15]. However, greater pennation angles are also associated with slower contraction velocities and thus lower power production [7,8,15]. For example, in previous studies from our laboratory, no significant correlations were found between vastus lateralis fiber pennation angle and performance in power-demanding activities, like sprinting, throwing, and jumping [12,13,14,16,17]. Muscle fascicle length has also been linked with performance in power-demanding activities [16,17,18,19,20,21], as longer fascicles allow for faster contraction speeds, a larger range of movement, and shift rightward the torque/angle relationship [7]. Indeed, sedentary individuals with increased power performance and sprinters seem to have longer muscle fascicles [22,23,24], and a greater percentage cross-sectional area (%CSA) of type II, especially of IIx muscle fibers [2,25], compared to individuals with lower power performance and endurance athletes, respectively. Interestingly, power performance may be better explained by the combination of muscle size, muscle architecture, and fiber composition, instead of using each one of these parameters alone [16]. Furthermore, the propagation velocity of action potentials along the sarcolemma of muscle fibers, which is associated with power performance, is also closely correlated with both type II muscle fiber %CSA and muscle fascicle length [26].

Taking into consideration all the above, it could be hypothesized that, for higher power performance, longer fascicle length and increased %CSAs of type II muscle fibers are required to be present in the same muscles. However, an earlier study in experimental animals (mice) showed no association between fiber type composition and fascicle length [27]. Strikingly, this correlation has not been examined in human muscle. Therefore, it is relevant to explore the possible relationship between muscle fiber composition and fascicle length in a particular muscle, and their contribution to human muscle power performance.

Muscle fiber composition and fascicle length may change in response to systematic training. Explosive resistance training results in increased fascicle length and the maintenance or decrease in the percentage of type IIx muscle fibers [28,29,30]. Therefore, the possible link between muscle fiber composition and fascicle length may be better revealed in individuals with minimal exposure to resistance/power training, yet with high-power potential. For this reason, female university students participated in this study, since this group is less exposed to strength/power training [26,27]. Thus, the aim of the present study was to investigate the relationship between vastus lateralis muscle fiber length and fiber type composition in individuals with minimal exposure to systematic resistance/power training. The underlying hypothesis was that such individuals may have muscles with long fascicles, together with a predominance of type II muscle fibers. This hypothesis suggests that in sedentary individuals, certain phenotypes should exist, by which muscles with longer fascicles may also be characterized by a proportional predominance of type II and %CSA (or vice versa).

## 2. Materials and Methods

### 2.1. Experimental Approach

**Figure 1:**
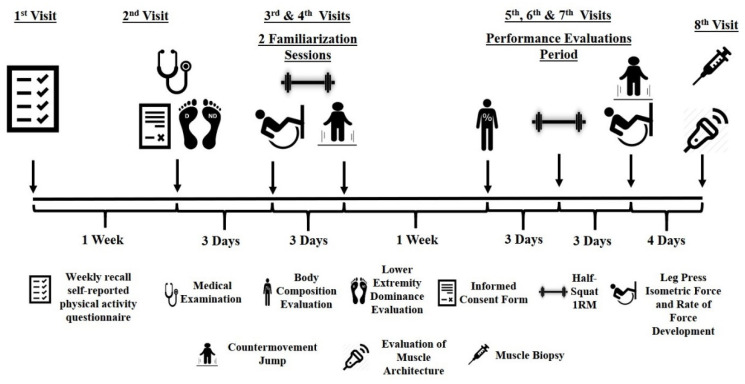
Schematic representation of the experimental protocol.

Participants were recruited via advertisements in university student societies. Responders visited the laboratory, where they completed a weekly recall self-reported physical activity questionnaire. Individuals who met the inclusion criteria (only healthy females, aged between 18–26, with no systematic experience in resistance training for at least 3 years) visited the laboratory for a second time, one week later, for a medical examination and evaluation of their lower extremity dominance (Waterloo footedness questionnaire; intraclass correlation coefficients (ICC) = 0.92), while they signed an informed consent form (Figure 1). Three days later they performed the first familiarization session with evaluations of power/strength performance (between tests a ten minutes rest was allowed). Three days later a second familiarization session was performed. One week after, body composition and strength/power performance were evaluated, on different occasions. At least two days after the final performance evaluations, the non-dominant leg’s vastus lateralis (VL) architecture was evaluated via ultrasonography while, 10 min later, muscle biopsies were obtained from the exact point of the ultrasonography evaluation.

### 2.2. Participants

Sixty (N = 60; age: 21.03 ± 2.1 years, body weight: 59.8 ± 9.7 kg, body height: 166.2 ± 6.5 cm) healthy female university students (power analysis: 0.921; G*Power ver. 3.1; FrankFaul, Universitat Kiel, Kiel, Germany) completed the experimental procedures (Table 1). All procedures were in accordance with the Declaration of Helsinki and approved by the local university ethics committee (ref. number 1013/25/5/2017), while all participants signed a written informed consent before entering the research program.

### 2.3. Procedures

#### 2.3.1. Evaluation of Body Composition

A total body scan was performed with dual energy x-ray absorptiometry (DPX-L, LUNAR Radiation, Madison, WI, USA), following the same methodology performed in previous studies from our laboratory [2,16,17,28,31,32]. All measurements were analyzed using the LUNAR radiation body composition program. Fat mass and lean body mass (LBM) were determined for the total body, as well as for the lower extremities. ICC for lower extremities LBM = 0.98, (95% CI: Lower = 0.95, Upper = 0.99), total LBM = 0.93, (95% CI: Lower = 0.89, Upper = 0.97), total % fat = 0.90, (95% CI: Lower = 0.85, Upper = 0.96), and lower extremities % fat = 0.94, (95% CI: Lower = 0.88, Upper = 0.98), *p* < 0.0001.

#### 2.3.2. Evaluation of Muscle Strength and Power

Leg Press Isometric Force and Rate of Force Development. Leg press isometric force and rate of force development were assessed according to the methodology previously described [10,17,28,30,33,34]. After reporting to the laboratory, participants were initially warmed up for 5 min on a stationary bicycle at 50 W. Participants were seated on a custom-made steel leg press chair and placed both feet on a force platform (Applied Measurements Ltd. Co., Aldermaston, UK, WPX0606-1000, sampling frequency 1000 Hz), which was positioned perpendicularly on a concrete laboratory wall. Data from the force platform were recorded and analyzed (Kyowa sensor interface PCD-320A). Knee angle was set at 120°, and hip angle was set at 100°. All participants were instructed to apply their maximum force as fast as possible for 3 s. Initially, participants performed 2 sub-maximal attempts and then 3 maximal attempts, with a 3-min rest between them. Participants were vocally encouraged to perform their best. Real-time visual feedback of the force applied was provided for each effort via a computer monitor placed just above the force platform. Variables calculated from the force–time curve included the maximum isometric force (MIF) and RFD. Maximum isometric force (as the highest peak on the force curve) and RFD at 20, 80, 100, 150, 200, and 250 ms, were calculated according to the following equation: RFD (N∙s^−1^) = ΔForce × ΔTime^−1^ [35,36]. The best performance according to the RFD at 150 ms was further used in statistical analyses. The ICC for MIF and RFD were: ICC = 0.90, (95% CI: Lower = 0.86, Upper = 0.96) and ICC = 0.92, (95% CI: Lower = 0.80, Upper = 0.98), respectively.

Jumping Performance. Jumping performance was evaluated with a countermovement jump test, according to the methodology previously described [2,10,28,31,33,37]. The test was performed on a force platform (Applied Measurements Ltd. Co. UK, WP800-1000 kg, 80 × 80 cm, sampling frequency 1 kHz) after the end of the isometric leg press evaluation. Subsequently, they performed 3 CMJs with submaximal intensity and then 3 maximal CMJs jumps, with a 2-min rest between each jump, with arms akimbo. All efforts were recorded and analyzed (Kyowa sensor interface PCD-320A) in order to calculate the following variables: (Jump height (cm) = ((0.5 × flight time)^2^ × 2^−1^) × 9.81 and (Maximum Power (W) = (body weight + Fmax) × 9.81 × flight time). The signal was filtered using a secondary low pass Butterworth filter with a cutoff frequency of 10 Hz. The best performance according to the jump power was used for further analysis. The ICCs for jump height and power were 0.87 (95% CI: Lower = 0.83, Upper = 0.95) and 0.91 (95% CI: Lower = 0.90, Upper = 0.99).

1-RM Half Squat Strength. This test was performed according to the methodology previously described [10,28,32,33,38]. Thirty minutes after the end of the CMJ testing, maximal half-squat strength was assessed in a Smith squat rack. Initially, participants performed 2–3 warm-up half-squat sets of 6–8 repetitions in a Smith machine with increasing loads. After that, they performed incremental submaximal efforts, with a 3-min rest between them, until they were unable to lift a heavier load. Knee bending was allowed at 90°. In all cases, two of the authors were present and vocally encouraged the participant in each trial. An adjustable iron rack was placed in the Smith machine to restrict the knee from bending under 90°. The ICC for evaluating 1-RM strength ranged from 0.920 to 0.980.

#### 2.3.3. Evaluation of Muscle Architecture

**Figure 2:**
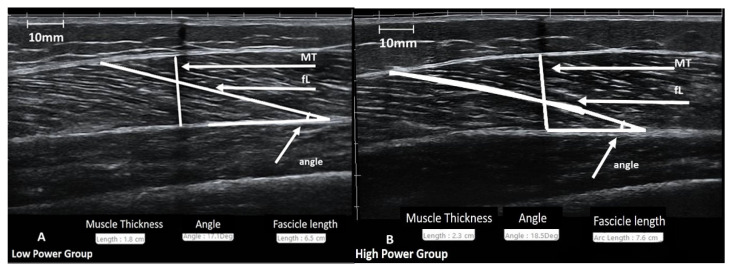
Representative images from the ultrasographic evaluation of vastus lateralis muscle for the Low-Power (**A**) and High-Power groups (**B**).

The procedure was performed according to the methodology followed in our laboratory [10,16,17,30,31,39]. All ultrasound images were obtained during the morning hours. Subjects remained at a prone position on the examination bed for at least 20 min before the ultrasound imaging. B-mode axial-plane ultrasound images (Product model Z5, Shenzhen Mindray Bio-Medical Electronics Co., Ltd., Shenzhen, China) were taken with a 10 MHz linear-array probe (38-mm width) with an extended-field-of-view (EFOV) mode. Ultrasound images were obtained at 50% of the distance from the central palpable point of the greater trochanter to the lateral condyle of the femur using the EFOV mode. Minimal pressure was applied to the skin to prevent any alteration of the underlying tissue image due to pressure. Self-adhesive paper was placed on the skin at the 50% point as a marker (image shadowing). The transducer was placed longitudinally on the femur, oriented parallel to the muscle fascicles and perpendicular to the skin. Based on this orientation, a dashed line (~10 cm) was drawn on the left and the right of the 50% point to identify and capture the largest continuous fascicle visualization. To obtain the muscle image, a continuous single view was taken by moving the probe along the marked dashed line. Additionally, the mediolateral angle of the probe was changed so that it remained perpendicular to the skin. Images was obtained from the vastus lateralis of the non-dominant lower limb. Muscle thickness was defined as the distance between the superficial and deep aponeurosis and was analyzed at the exact point at 50% (Figure 2). Fascicle angle was defined as the angle of insertion of muscle fascicles into the deep aponeurosis. Fascicle length was defined as the fascicular path between the insertion of the fascicle into the upper and deeper aponeurosis. For each image, a visually clear fascicle was chosen to be analyzed for its angle and length. All images were analyzed using image analysis software (Motic Images Plus, 2.0, Hong Kong, China). The ICC for this method had been recently tested [10], and it was 0.97 (95% CI: 0.87–0.99, *p* = 0.001) for muscle thickness, 0.88 (95% CI: 0.60–0.97, *p* = 0.001) for fascicle angle, and 0.84 (95% CI: 0.47–0.96, *p* = 0.001) for fascicle length.

#### 2.3.4. Muscle Biopsies and Histochemistry

The procedure was done according to previous reports from our laboratory [2,28,30,33,37,38,39]. Muscle samples were obtained with Bergström needles, from the middle part of the non-dominant lower extremity vastus lateralis, at the exact point of ultrasound imaging, and under local anesthesia by a trained physician. Samples were aligned, placed in an embedding compound, and frozen in isopentane pre-cooled to its freezing point, and subsequently stored in liquid nitrogen until analysis. Serial cross-sections of 10 μm thick were cut at −20 °C and stained for myofibrillar ATPase after pre-incubation at pH 4.3, 4.6, and 10.3. A mean of 435 ± 120 muscle fibers from each participant were classified as type I, IIa, or IIx. The CSA and percentage cross-sectional area (%CSA) of all the classified muscle fibers were measured with an image analysis system (Image Pro, Media Cybemetics Inc., Silver Spring, MD, USA) at a known and calibrated magnification. The ICCs for the percentage of type I, IIa, and IIx fibers in our laboratory were 0.96, 0.95, and 0.93, respectively (95% CI: Lower = 0.91, 0.92, 0.87, and Upper = 0.99, 0.98, 0.95, respectively).

### 2.4. Statistical Analyses

For the purpose of the present study, initially, all participants were analyzed as one group, while for the second part, they were assigned into two groups (High Power (HP) N = 30, Low Power (LP) N = 30), according to their power performance (cutting point: median of the countermovement jump power). All data are presented as mean and standard deviation (±SD). A Shapiro–Wilks test was conducted to test the normality of the data. No violations of distribution normality were found. An independent samples t-test was conducted to compare the differences between the 2 groups. Calculation of effect size (η^2^) was also performed. Pearson’s (r) product–moment correlation coefficients were computed to explore the relationships between variables. The interpretation of the observed correlations was performed according to Hopkins’ ranking: correlations coefficients between 0.3 and 0.5 were considered moderate, between 0.51 and 0.70 large, between 0.71 and 0.90 very large, and >0.91 almost perfect. Multiple linear regression analyses were also performed. *P* ≤ 0.05 was used as a 2-tailed level of significance. Statistical analyses were performed with SPSS Statistics Ver. 23.0 (IBM Corporation, Chicago, IL, USA).

## 3. Results

### 3.1. Comparison between Groups

Significant differences were found between participants of the High- and Low-Power groups, for total and lower extremity LBM, VL thickness, fascicle length, and fiber type composition (η^2^: 0.119–0.239; *p* < 0.01; Table 1). Significant differences between the two groups were found in all performance tests (η^2^: 0.082–0.598; *p* < 0.05; Table 1).

### 3.2. Correlations between Biological and Performance Parameters

When all participants were considered as one group (N = 60), significant correlations were found between lower extremity LBM, VL thickness–pennation angle and CMJ power, MIF, RFD after 150 ms from the onset of muscle contraction, and half squat maximum strength (r: 0.267–0.595; *p* < 0.01; Table 2 and Supplementary Material Table S1). VL muscle fiber CSA and %CSA were only related to MIF and half squat 1RM (r: −0.500–0.509; *p* < 0.01). When these correlations were investigated in each group separately, higher correlations were observed in the High-Power group (r: −0.672–0.645; *p* < 0.001; Table 2). In contrast, the only significant correlations that were found in the Low-Power group were found between lower extremity LBM and all performance variables (r: 0.312–0.444; *p* < 0.05; Table 2), as well as between VL thickness and CMJ power and MIF (r: 0.366 and 0.290 respectively; *p* < 0.05). Multiple linear regression analyses revealed that CMJ power (R^2^: 0.720, *p* < 0.001), MIF (R^2^: 0.692, *p* < 0.001), and half squat 1RM (R^2^: 0.632, *p* < 0.001) could be better explained by the combination of lower extremity LBM, VL fascicle length, type IIa, IIx muscle fiber %CSA (Table 3).

### 3.3. Correlations between Vastus Lateralis Architecture and Muscle Fiber Compostition

**Figure 3:**
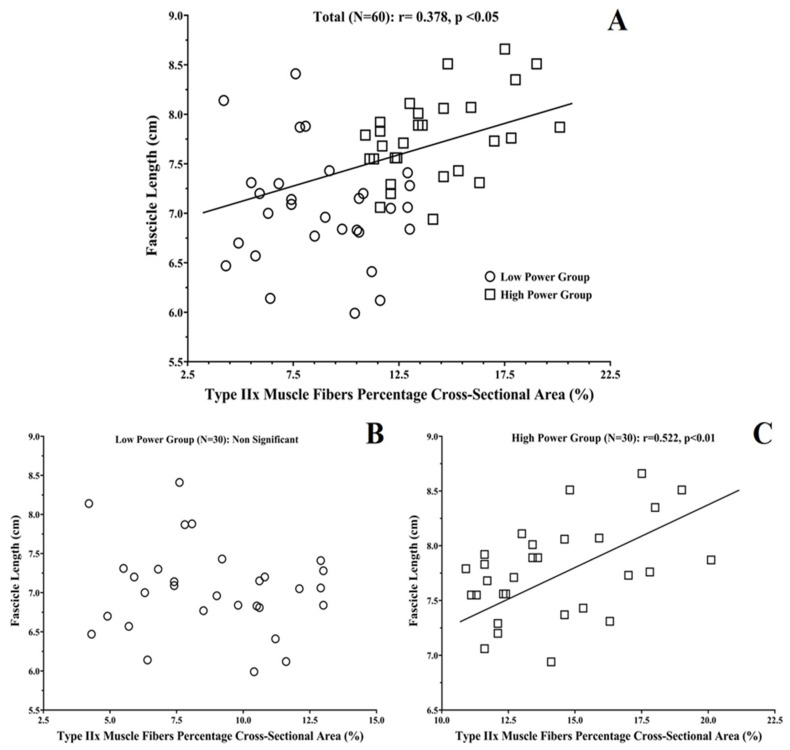
Correlation plots between vastus lateralis percentage cross-sectional area of type IIx muscle fibers and fascicle length for: (**A**) all participants as one group (N = 60), and (**B**) Low-Power (N = 30) and (**C**) High-Power Groups (N = 30).

Type IIa and IIx fiber CSAs were related to muscle thickness (r: 0.312 and 0.294 respectively; *p* < 0.05). Type I muscle fiber %CSA was negatively related to muscle thickness and fascicle length (*p* < 0.05; Table 4). Positive correlations were found between type IIa, IIx muscle fiber %CSA and muscle thickness, pennation angle, and fascicle length (r: 0.300–0.378; *p* < 0.05). All these correlations were stronger in the High-Power group (r: −0.500–0.523; *p* < 0.05; Table 4 and Figure 3). No significant correlations were found for muscle fiber proportion and muscle architecture in the Low-Power group (*p* > 0.05).

## 4. Discussion

The current results do not support the hypothesis of a strong link between the percentage of each muscle fiber type and muscle fascicle length in the vastus lateralis in young females. This suggests that fibers expressing fast contracting proteins are not necessarily longer, at least in the vastus lateralis of non-strength/power-trained young females. Fascicles detected with B-mode ultrasonography are thought to represent bundles of fibers that presumably contain a mosaic of type I and type II fibers. Even if these bundles increase in length in response to exercise training [10,29,30], this elongation would presumably be comparable for all the fibers in the bundle, both type I and II fibers, suggesting a dissociation of the fiber composition and fascicle length, which justifies the lack of correlation found here between these two parameters. Nevertheless, this rationale is based on the assumption that all the fibers in a bundle run its full length, which has been shown not to be true, at least in animal muscles [40]. However, even if all the fibers in a bundle run its full length in human muscles, it is unlikely that a muscle bundle would be composed of only a certain type of fiber (e.g., type II), since such a fiber type grouping has only been shown in neuromuscular disease or aged muscles [41].

The absence of any significant correlation between muscle fiber type proportion and muscle fascicle length was somewhat expected; according to the results of an earlier study in experimental animals [17]. In addition, the mechanical properties of each muscle, and thus power production, seem to depend primarily on the combination of fiber type distribution and size, e.g., %CSA, and not only on the number of type II fibers [26,42,43,44]. For example, it has been reported that in a world champion shot putter, with a predominance of type I muscle fibers (~60%), his hypertrophied type II muscle fibers occupied 67% of VL the area, making him able to achieve greater power and shot put performance, compared to another shot-putter with a predominance of type II fibers but with smaller type II fiber CSA [45]. Additionally, changes in the %CSA of muscle fibers seem to be linked with the accompanying changes in power performance [28,38,44,46]. The physiological background of muscle fiber %CSA importance in power performance is based on both, the properties of the large fibers, and of the mechanical characteristics of each fiber type. In healthy, non-trained, young human muscles, large fibers contain more myofibrils and myosin-actin filaments in a parallel order, and thus a higher number of active cross-bridges that work together during a contraction, leading to increased absolute and relative peak power compared to fibers with smaller diameters [47,48,49,50]. Type IIa, and especially IIx muscle fibers, due to their specific types of myosin heavy/light chains, and their biochemical properties, like the ATPase kinetics etc [3,48,51], have faster cross-bridge cycle rates, shorter contraction times, greater contraction velocities, and thus they are able to produce greater forces/power compared to type I muscle fibers [52,53]. Thus, individuals having higher %CSAs of type II, and especially of IIx muscle fibers, have a greater power capacity. Longer fascicles, due to the increased number of sarcomeres in series that they theoretically have, are characterized by increased shortening velocities, force–velocity curve, a wider force-length curve, and thus by higher force/power outputs compared to short fascicles [54]. However, if two muscles with identical fascicle length and pennation angles have different PCSAs, probably because of diverse fiber type compositions and size, the muscle with greater CSA will be able to induce increased amounts of force and power, due to the greater force–velocity/length curves that characterize it [55]. Indeed, only when the cross-sectional area of muscle fibers was also considered were there low to moderate correlations between muscle fiber %CSA and fascicle length.

When the cross-sectional area of muscle fibers was also considered, there was a stronger correlation between type II muscle fibers and fascicle length for the participants with higher power output. It seems that the participants with thicker type II muscle fibers were more powerful, and this was accompanied by longer fascicles. This suggests that the current participants with higher power performance were characterized by longer fascicles and larger type II fiber cross-sectional areas, perhaps due to genetic predisposition. The absence of any significant correlations between VL architecture parameters, fiber type composition, and power performance in the Low-Power group, and the greater correlations that were found between these factors in the High-Power group, was supported by the results of previous studies, reporting that these biological factors are crucial determinant parameters of performance only in participants with an increased training background and/or power performance [16,35,56,57]. In addition, these results indicate that the great interindividual variability of power performance that exists, even between non-trained participants [35,36,56], is due to the variation of physiological phenotypes related to the length and fiber composition of muscle fascicles, with individuals achieving greater power production having more muscle mass, type II muscle fiber %CSA, and longer fascicle compared to sedentary participants with lower performance in power/strength-demanding activities. However, it has been recently reported that the variance of RFD performance between young recreationally active men, could be explained by the individuals’ maximal motor unit discharge rates and recruitment intervals [58,59]. As a final point, the lean body mass of the lower extremities seems to be the most important contributor of CMJ power and maximum isometric/dynamic maximum strength, while muscle fiber type composition and muscle architecture of the vastus lateralis have a lower importance, as has been previously reported in sedentary [26] and novice power-trained males [16].

In conclusion, vastus lateralis fascicle length is moderately associated with the percentage area of muscle occupied by type IIa and IIx muscle fibers. These correlations are more pronounced in those non-trained females with a higher ability for power production and greater lower extremity lean body mass. The current results do not provide direct evidence that longer fascicles are composed by type IIa and IIx muscle fibers. Rather they indicate that the most powerful, non-trained females, with longer VL fascicle lengths are also characterized by an increased Type II %CSA. Finally, it seems that power production is an outcome of the synergistic contribution of the legs’ LBM, muscle fascicle length, and type IIa and IIx muscle fibers %CSA; and it may not be fully explained by each of the previously mentioned biological parameters alone. Whether these conclusions are also true in experienced/trained female athletes and in male participants should be verified in future studies.

## Figures and Tables

**Table 1:** Participants’ lean body mass, vastus lateralis architecture, muscle fiber composition, and power performance.

		All Participants (N = 60)	Comparison between the Groups		
			High Power (N = 30)	Low Power (N = 30)	Partial η^2^/*p* Values
Lean Mass (kg)		41.7 ± 4.2	43.4 ± 4.2	39.9 ± 3.5	0.173/0.009 *
Legs Lean Mass (kg)		14.5 ± 1.6	15.3 ± 1.4	13.6 ± 1.5	0.239/0.002 *
**Vastus Lateralis Architecture**					
Muscle Thickness (cm)		2.1 ± 0.3	2.2 ± 03	1.8 ± 0.2	0.157/0.038 *
Pennation angle (°)		17.7 ± 3.7	18.4 ± 3	17.1 ± 3.2	0.029/0.192
Fascicle length (cm)		7.1 ± 0.7	7.3 ± 0.7	6.5 ± 0.8	0.181/0.001 *
**Vastus Lateralis Fiber Type Composition**					
Type I Percentage (%)		48.65 ± 6.4	46.1 ± 7.8	51.2 ± 4.6	0.126/0.009 *
Type IIa Percentage (%)		38.5 ± 6.7	37.6 ± 8.0	39.3 ± 5.2	0.016/0.337
Type IIx Percentage (%)		12.9 ± 5.1	16.3 ± 5.8	9.5 ± 3.4	0.127/0.005 *
Type I Cross-Sectional Area (μm^2^)		3349 ± 444	3622 ± 471	3173 ± 425	0.123/0.010 *
Type IIa Cross-Sectional Area (μm^2^)		3411 ± 462	3803 ± 420	3219 ± 505	0.119/0.010 *
Type IIx Cross-Sectional Area (μm^2^)		2627 ± 431	2997 ± 231	2162 ± 229	0.124/0.009 *
Type I Percentage Cross-Sectional Area (%)		47.9 ± 7.5	43.6 ± 8.9	53.1 ± 5.9	0.128/0.009 *
Type IIa Percentage Cross-Sectional Area (%)		40.7 ± 7.2	42.6 ± 8.2	38.7 ± 6.0	0.092/0.043 *
Type IIx Percentage Cross-Sectional Area (%)		11.4 ± 4.4	14.2 ± 4.8	8.2 ± 3.9	0.136/0.002 *
**Counter Movement Jump**					
Power (W)		1948 ± 388	2236 ± 315	1640 ± 145	0.598/0.000 *
Height (cm)		25.6 ± 4.2	27.1 ± 4.8	24.1 ± 2.7	0.134/0.005 *
**Maximum Isometric Strength and Rate of Force Development**					
Maximum Isometric Force (N)		2035 ± 607	2215 ± 697	1849 ± 435	0.092/0.019 *
Rate of Force Development (N·s^−1^)	20 ms	6799 ± 2644	8030 ± 2450	5526 ± 2227	0.227/<0.001 *
	80 ms	8777 ± 3228	10347 ± 2800	7153 ± 2844	0.248/<0.001 *
	100 ms	8740 ± 3028	10192 ± 2593	7238 ± 2727	0.241/<0.001 *
	150 ms	8025 ± 2382	9070 ± 2145	6944 ± 2147	0.202/<0.001 *
	200 ms	7018 ± 1952	7779 ± 1730	6231 ± 1878	0.159/0.002 *
	250 ms	6080 ± 1660	6663 ± 1491	5478 ± 1632	0.129/0.005 *
**Maximum Strength**					
Half Squat 1-RM Strength (kg)		112.7 ± 19.7	117.6 ± 20.5	107.8 ± 17.7	0.082/0.043 *

Values are represented as Mean ± SD. With (*) denoting the significant differences between the 2 groups (High power, Low power).

**Table 2:** Correlations between lower extremity lean body mass, vastus lateralis architecture, fiber type composition, and performance variables for the total participants, as well as for each group separately. Only significant correlations are presented (*p* < 0.05).

		Legs Lean Body Mass	Vastus Lateralis Architecture			Vastus Lateralis Fiber Type Composition							
						Percentage		Cross-Sectional Area			Percentage Cross-Sectional Area		
			Muscle Thickness	Pennation Angle	Fascicle Length	IIa	IIx	I	IIa	IIx	I	IIa	IIx
**All Participants (N = 60)**													
CMJP		0.595	0.411	0.279	0.341								
MIF		0.389	0.330		0.355			−0.403	0.410	0.384	−0.499	0.434	
RFD	150 ms	0.456		0.282									
	200 ms	0.432	0.274	0.306									
	250 ms	0.447	0.303	0.316	0.372								
1RM		0.426	0.267	0.300		0.407		−0.487	0.511	0.509	−0.500	0.512	
**High-Power Group (N = 30)**													
CMJP		0.645	0.477	0.320	0.400	0.497	0.466					0.500	0.421
MIF		0.500	0.480		0.499			−0.575	0.574	0.538	−0.672	0.624	0.703
RFD	150 ms	0.514		0.347	0.324								
	200 ms	0.537	0.333	0.324	0.375								
	250 ms	0.567	0.378	0.360	0.400								
1RM		0.612	0.389	0.500		0.577	0.451	−0.598	0.630	0.599	−0.601	0.621	0.579
**Low-Power Group (N = 30)**													
CMJP		0.444	0.366										
MIF		0.312	0.290										
RFD	250 ms	0.399											
1RM		0.326											

CMJP: Countermovement Jump Max Power; MIF: Max Isometric Force; RFD: Rate of Force Development; 1RM: Half Squat Maximum Strength.

**Table 3:** Results from multiple linear regression analyses. Beta coefficients (B) were used as indicators of lower extremity lean body mass, vastus lateralis fascicle length, type IIa, and IIx percentage cross-sectional area relative strengths for the determination of power performance (Only significant coefficients are presented, N = 60).

Performance Parameter (R^2^/p)		Lower Extremities Lean Body Mass	Fascicle Length	Type IIa Percentage Cross-Sectional Area	Type IIx Percentage Cross-Sectional Area
Countermovement Jump Max Power (0.720/<0.001)	**B**	0.621	0.481	0.384	0.548
	***p***	<0.001	0.002	0.029	0.004
Max Isometric Force (0.692/<0.001)	**B**	0.589	0.424	0.625	0.384
	***p***	<0.001	<0.001	<0.001	0.013
Half Squat Maximum Strength (0.632/<0.001)	**B**	0.712	0.399	0.521	0.412
	***p***	<0.001	0.011	<0.001	<0.001

B: Standardized Beta coefficient of linear regression analysis.

**Table 4:** Correlations between vastus lateralis architecture and fiber type composition, for the all participants, as well as for each group separately. Only significant correlations are presented (*p* < 0.05).

	Vastuls Lateralis Fiber Type Composition					
	Cross Sectional Area			Percentage Cross-Sectional Area		
Vastuls Lateralis Architecture	I	IIa	IIx	I	IIa	IIx
**All Participants (N = 60)**						
Muscle Thickness		0.312	0.294	−0.400	0.368	0.325
Pennation Angle		0.300	0.314		0.322	0.300
Fascicle Length				−0.333	0.321	0.378
**High Power Group (N = 30)**						
Muscle Thickness		0.425	0.471	−0.500	0.523	0.500
Pennation Angle		0.392	0.400		0.491	0.420
Fascicle Length				−0.414	0.499	0.522

CSA: Cross sectional area, %CSA: Percentage cross sectional area of muscle occupied by muscle fiber.

## Data Availability

Data available after a reasonable request from the authors.

## Supplementary Materials

The following are available online at https://www.mdpi.com/article/10.3390/sports9050056/s1, Table S1: Correlations between lower extremities lean body mass, vastus lateralis architecture, fiber type composition, and performance variables, for total participants, as well as for each group separately.
