# Supplementary material for: Weak Association between Vastus Lateralis Muscle Fiber Composition and Fascicle Length in Young Untrained Females
Source: Sports (Basel). 2021 Apr 28;9(5):56. doi: 10.3390/sports9050056 (PMC8146508; doi:10.3390/sports9050056)
Supplement: Supplementary file 1 [file sports-09-00056-s001.zip › sports-1117903-supplementary.pdf]

Table S1. Correlations between lower extremities' lean body mass, Vastus Lateralis' architecture, fiber type composition and performance variables, for the total of participants, as well as for each group separately

		Legs Lean Body Mass	Vastus Lateralis Architecture			Vastus Lateralis Fiber Type Composition								
						Percentage			Cross-Sectional Area			Percentage Cross-Sectional Area		
			Muscle Thickness	Pennation Angle	Fascicle Length	I	IIa	IIx	I	IIa	IIx	I	IIa	IIx
**All Participants (N = 60)**														
CMJP		0.595 *	0.411 *	0.279 *	0.341 *	−0.123	0.067	0.047	−0.069	0.079	0.238	0.028	−0.010	−0.031
MIF		0.389 *	0.330 *	0.198	0.355 *	−0.056	0.071	−0.025	−0.403 *	0.410 *	0.384 *	−0.499 *	0.434 *	0.221
RFD	20 ms	0.365 *	0.133	0.145	0.224	0.031	−0.072	0.058	0.047	0.151	0.206	0.151	−0.094	−0.097
	80 ms	0.433 *	0.161	0.204	0.248	0.001	−0.076	0.097	0.069	0.203	0.183	0.150	−0.107	−0.072
	100 ms	0.423 *	0.189	0.224	0.255	0.013	−0.073	0.078	0.071	0.231	0.197	0.144	−0.093	−0.086
	150 ms	0.456 *	0.248	0.282 *	0.245	0.045	−0.052	0.014	0.080	0.248	0.246	0.112	−0.038	−0.124
	200 ms	0.432 *	0.274 *	0.306 *	0.253	0.090	−0.053	−0.041	0.015	0.311	0.190	0.115	−0.009	−0.177
	250 ms	0.447 *	0.303 *	0.316 *	0.372 *	0.092	−0.027	−0.076	−0.045	0.322	0.168	0.091	0.034	−0.206
1RM		0.426 *	0.267 *	0.300 *	−0.003	0.108	0.407 *	0.208	−0.487 *	0.511 *	0.509 *	−0.500 *	0.512 *	0.230
**High Power Group (N = 30)**														
CMJP		0.645 *	0.477 *	0.320 *	0.400 *	−0.117	0.497 *	0.466 *	−0.039	0.200	0.097	−0.076	0.500 *	0.421 *
MIF		0.500 *	0.480 *	0.298	0.499 *	−0.039	0.150	0.089	−0.575 *	0.574 *	0.538 *	−0.672 *	0.624 *	0.703 *
RFD	20 ms	0.111	0.094	0.100	0.148	−0.103	0.169	0.072	0.279	0.242	0.236	0.156	0.227	0.072
	80 ms	0.230	0.114	0.145	0.076	−0.104	0.117	0.006	0.245	0.284	0.181	0.171	0.271	0.024
	100 ms	0.235	0.174	0.200	0.086	−0.103	0.134	0.016	0.252	0.330	0.214	0.174	0.262	0.045
	150 ms	0.514 *	0.319	0.347 *	0.324 *	−0.016	0.139	0.139	0.238	0.305	0.325	0.116	0.232	0.148
	200 ms	0.537 *	0.333 *	0.324 *	0.375 *	−0.017	0.182	0.090	0.274	0.365	0.284	0.132	0.276	0.123
	250 ms	0.567 *	0.378 *	0.360 *	0.400 *	−0.025	0.164	0.102	0.359	0.388	0.296	0.084	0.346	0.146
1RM		0.612 *	0.389 *	0.500 *	0.245	−0.245	0.577 *	0.451 *	−0.598 *	0.630 *	0.599 *	−0.601 *	0.621 *	0.579 *
**Low Power Group (N = 30)**														
CMJP		0.444 *	0.366 *	0.133	0.031	0.248	−0.125	−0.141	−0.144	−0.117	0.295	0.269	−0.065	−0.280
MIF		0.312 *	0.290 *	0.087	0.215	0.030	0.048	−0.117	−0.132	0.191	0.003	0.059	0.117	−0.266
RFD	20 ms	0.401	−0.068	0.057	0.134	−0.001	0.170	−0.255	−0.155	0.112	0.085	0.112	0.137	−0.349
	80 ms	0.393	−0.011	0.139	0.122	0.020	0.149	−0.266	−0.139	0.192	0.090	0.095	0.144	−0.342

	100 ms	0.398	−0.009	0.144	0.124	0.028	0.145	−0.273	−0.138	0.212	0.095	0.077	0.161	−0.307
	150 ms	0.224	−0.038	0.142	0.102	0.091	0.100	−0.219	−0.139	0.238	0.098	0.064	0.140	−0.330
	200 ms	0.323	0.003	0.204	0.129	0.099	0.090	−0.227	−0.203	0.240	0.036	0.060	0.159	−0.339
	250 ms	0.399 *	0.030	0.220	0.159	0.122	0.089	−0.224	0.244	0.234	−0.013	0.064	0.164	−0.343
1RM		0.326 *	0.121	0.208	−0.249	0.212	−0.269	0.116	−0.293	−0.361	0.082	0.049	−0.085	0.056

With (*) denoted the significant correlations ($p < 0.05$).
